# Tensile and Structural Performance of Annealed 3D-Printed Polymer Composite Impellers for Pump-as-Turbine Applications in District Heating Networks

**DOI:** 10.3390/ma19010127

**Published:** 2025-12-30

**Authors:** Dominik Błoński, Grzegorz Romanik, Michał Augustyn, Paweł Regucki

**Affiliations:** Department of Energy Conversion Engineering, Faculty of Mechanical and Power Engineering, Wrocław University of Science and Technology, 27 Wybrzeze Wyspianskiego Street, 50-370 Wroclaw, Poland; grzegorz.romanik@pwr.edu.pl (G.R.); michal.augustyn.a@gmail.com (M.A.); pawel.regucki@pwr.edu.pl (P.R.)

**Keywords:** 3D printing, additive manufacturing, 3D printing annealing, pump as a turbine

## Abstract

This study investigates the mechanical performance of three temperature-resistant 3D-printable polymer composites for turbine impellers used in district heating networks for pressure reduction. Using fused deposition modeling (FDM), tensile strength and deformation of ASA-X CF10, PA6-GF30, and ePAHT-CF15 were evaluated at temperatures representative of real operating conditions (60–130 °C). These polymer composites were systematically tested, with particular emphasis on annealed ePAHT-CF15. Results demonstrated that annealing significantly improved mechanical performance, yielding higher tensile strength, Young’s modulus, and reduced deformation. Structural analyses confirmed that ePAHT-CF15, particularly when annealed at 200 °C, exhibited superior thermal stability and rigidity, making it the optimal material choice for high-temperature turbine impeller applications. These findings support the design of 3D-printed composite impellers for pump-as-turbine applications in district heating systems, where high stiffness and heat resistance are required.

## 1. Introduction

This work aimed to enhance pump operation in reverse mode by integrating rapid prototyping techniques. Pumps acting as turbines offer a cost-effective solution for energy recovery in heating districts, as replacing pressure control valves with turbines can significantly improve energy efficiency. However, pumps used as turbines tend to be less efficient than custom-designed turbines. To address this, replacing the pump impeller with a turbine impeller can boost efficiency. The most economical way to create a new impeller design is through FDM 3D printing technology.

A key consideration for 3D-printed impellers, especially in high-temperature environments like water heating systems, is the strength and material properties. The selected material must maintain structural integrity with minimal distortion during large-volume printing. This is critical for accurate geometry reproduction and to ensure that the impeller performs reliably. The goal is to select proper material for manufacturing an impeller that can be printed on mid-range 3D printers, without the need for an enclosure, while still ensuring high strength and durability during operation at elevated temperatures.

Apart from material strength testing, this research also included a thorough flow analysis to determine the forces acting on the impeller, followed by a structural analysis to evaluate the stress and strain experienced by the 3D-printed impeller. A particular focus is placed on minimizing deformations and maintaining optimal force distribution during operation. In order to sustain the turbine’s high volumetric efficiency, it is critical to maintain small gaps at the axial and radial seals. However, increased temperature reduces the Young’s modulus, reducing the material’s stiffness. Excessive deformation of the impeller walls under operational forces can lead to contact with the pump axial seals or volute, resulting in energy losses. To mitigate this, increasing the clearance between the impeller and stationary parts may prevent such contact; however, this would also introduce leakage, thereby compromising the overall efficiency of the flow path system.

Three-dimensional printing is a rapidly developing manufacturing technology that is gaining popularity in many industries and scientific applications thanks to several key factors:

- Design flexibility: The ability to create custom and complex shapes without the need for expensive molds;
- Prototyping speed: Reducing the time needed to transform an idea into a physical model;
- Cost reduction: Lower material consumption and reduced production waste.

It is important to note that 3D printing can align with current environmental trends. This technology reduces waste because materials are used only where needed. Furthermore, new biodegradable materials are being developed, which can reduce the negative impact on the environment.

When designing machine components, operating temperature must be taken into account, especially when replacing metal with polymers. Components operating at water temperatures up to 120 °C require enhanced mechanical strength and stiffness. To strengthen 3D-printed machine elements subjected to demanding operational conditions, one should consider the following approaches [1,2,3,4,5]:

- Material Modification: Incorporating additives or blending with other materials (like nylon, carbon fiber, or other polymers) to improve strength;
- Optimized Printing Parameters: Adjusting print settings such as layer height, infill density, print temperature, and print speed to produce stronger parts;
- Post-Processing Techniques: Methods like annealing (heat treatment) can increase crystallinity, resulting in higher strength and heat resistance;
- Using Higher-Quality Filament: Selecting premium filaments that have better fiber alignment and fewer impurities can lead to stronger printed parts.

The scientific literature presents a variety of approaches aimed at enhancing the properties of 3D-printed structures and porous materials. A recurring theme is the influence of impregnation and precipitation processes on the physical and mechanical characteristics of these materials. Notably, the impregnation of porous structures with various solutions can significantly modify parameters such as strength, durability, resistance to biological degradation, and thermal stability.

The 3D printable polymers, such as Nylons, are prone to water absorption. Our laboratory tests indicate that the specimens can absorb up to about 14 wt.% water after 15 days of soaking. As a result, dimensional changes of the 3D-printed parts are inevitable. In addition to operation at elevated temperatures, moisture uptake is therefore a key concern for this technology. It is therefore worth mentioning recent advances in the impregnation of porous materials.

Recent advances in the field of environmentally friendly wood impregnation using inorganic salts such as Na_2_SiO_3_, which improve wood properties such as mechanical strength, durability, and fire resistance, have been widely described in many publications. Study [6] describes CO_2_ stabilization under pressure and temperature to enhance chemical and physical properties. Research [7] compares the respiratory impregnation method and methods with the vacuum progressive impregnation method showing better penetration and improvements in dimensional stability and combustion behavior. Furuno and Imamura [8] use water glass-boron systems to create decay and termite-resistant wood via insoluble inorganic compounds. Research conducted by Zhou et al. [9] employs cyclic pressure methods with Na_2_SiF_6_ to boost leaching resistance and mechanical performance. Zhang et al. [10] reports that silicon precursors enhance mechanical and flame-retardant properties by depositing silica within cell walls. Ultrasonic-assisted sol-gel techniques [11] further increase silicon content, improving strength and thermal stability. Comparative analysis [12] indicates sodium silicate treatment outperforms phenol-formaldehyde, thanks to Si–O–Si bonds and hydrogen bonding, enhancing overall wood performance.

Another literature source [13] gives a description of finite element analysis to assess the stress distribution and structural integrity of plastic pump impellers, providing insights into failure mechanisms and design optimization. In the realm of additive manufacturing, reviews such as [14] highlight the potential of thermoplastic and polymer composites in rapid prototyping technologies, emphasizing the significance of fillers—both mineral and synthetic—in improving processability and material performance.

The authors’ experiments with impregnating 3D prints did not yield the anticipated enhancements in mechanical properties. Alongside chemical strengthening methods such as sodium silicate impregnation, post-processing techniques like heat treatment (annealing) have emerged as crucial strategies for enhancing the performance of thermoplastic components produced through material-extrusion 3D printing. Recent advances in polymer modification and post-processing techniques are exemplified by studies [15,16,17], which examine the effects of annealing on the tensile and thermal properties of 3D-printed polylactide (PLA) and polyamide (PA) composites. These investigations demonstrate that controlled annealing can enhance strength and thermal resistance while balancing dimensional stability. Article [18] describes the mineral and synthetic fillers used in 3D printing. Moreover, the integration of mineral fillers like silica nanoparticles into polymer matrices, as detailed in [19], has yielded significant improvements in mechanical and thermal properties, especially when surface-functionalized to promote covalent bonding with the polymer backbone. Finally, research [20,21] explores the influence of processing parameters—such as strain rate, moisture absorption, and impregnation techniques—on the mechanical performance of fiber-reinforced composites produced via additive manufacturing or traditional manufacturing methods. These findings underscore the importance of processing controls in achieving desired material characteristics. Annealing promotes molecular relaxation and recrystallization of semi-crystalline polymers, which leads to higher stiffness, yield strength, and heat-deflection temperature, while also reducing residual stresses from the printing process. For polyamide-based materials, Ferreira et al. showed that annealing of PA12 and short-fiber-reinforced PA12 FFF specimens significantly increases tensile and flexural strength and reduces anisotropy between print directions [22]. Similar improvements have been reported for PA6 and PA6-based composites, where controlled annealing increases crystallinity and tensile modulus but can also lower elongation at break [23,24,25]. More recent work on short carbon-fiber- and glass-fiber-reinforced nylons demonstrates that optimized annealing schedules, including multi-stage heat treatments, can enhance strength and dimensional stability without excessive warpage, which is critical for engineering parts produced by FDM [26,27,28,29].

In nylon composites for structural applications, annealing is often studied together with other design variables, such as fiber content, build orientation, and infill strategy. Belei et al. optimized process parameters for short-carbon-fiber-reinforced polyamide and showed that, when combined with appropriate post-heat treatment, high levels of stiffness and strength comparable to injection-molded materials can be achieved [30]. Xu et al. investigated carbon fiber addition to PA12 polyamide and its effect on the mechanical properties with the focus on anisotropy and fiber orientation [31]. Balan et al. analyzed post-heat treatment of PA12 produced by both material extrusion and selective laser sintering and confirmed that thermal post-processing improves tensile strength and reduces surface defects, which is particularly relevant for components exposed to elevated temperatures [32]. These studies indicate that for high-temperature service, such as pump and turbine components, nylon-based materials benefit strongly from annealing in terms of both mechanical performance and thermal stability.

A turbomachinery-oriented reference on additively manufactured polymer-composite impellers shows great progress in this technology. Zirak et al. reviewed polymeric and polymer-composite impeller fabrication routes, including additive manufacturing, and discussed the main application drivers (e.g., corrosion resistance and reduced cost) alongside key limitations such as service-temperature capability, erosion/abrasion resistance, and the need to validate structural integrity under realistic hydraulic loads. This review supports the motivation to couple material selection with application-specific mechanical verification for operational impellers [33].

Mansour et al. presented an engineering workflow for carbon-fiber-reinforced polymer impellers that combines tensile-property characterization, CFD-based pressure loading, and static FEM structural assessment to estimate allowable operating power and compare candidate composite impellers against conventional metallic materials. Their approach highlights how experimentally measured stiffness and strength can be propagated into a structural safety assessment under pump operating loads [34].

Papageorgiou et al. reported hydraulic performance testing and CFD assessment of a material-extrusion-printed composite impeller, showing that geometric redesign and composite fabrication can improve pump performance relative to a baseline cast-iron impeller. The study emphasizes that efficiency improvements must be demonstrated experimentally and interpreted together with manufacturing constraints and surface/geometry quality [35].

Raja et al. investigated process-parameter optimization for a composite thermoplastic impeller (graphene-reinforced PETG) and demonstrated that printing parameters measurably influence manufacturability and mechanical performance proxies. Although focused on a different polymer system, the study reinforces the broader point that robust impeller development requires controlled printing conditions and post-processing when targeting functional turbomachinery components. In parallel, turbomachinery-oriented AM studies report performance validation of composite impellers against baseline metal parts and highlight the importance of surface quality, material selection, and verification testing for end-use operation [36].

Motivated and built upon this extensive body of knowledge, the present study investigates the effects of annealing on 3D-printed samples. We aim to evaluate how these post-processing treatments influence mechanical strength, dimensional stability, and thermal resistance in additive manufacturing contexts and what is the material selection effect on mechanical performance of 3D-printed impeller.

## 2. Methodology for Tensile Testing of Temperature-Resistant Polymers

### 2.1. Filament Selection

This section outlines the methodology used to assess the ultimate tensile strength of three temperature-resistant polymers: ASA-X CF10, PA6-GF30 “Low warp”, and ePAHT-CF15 mady by Spectrum Filaments (Spectrum Group Sp. z o.o., Pecice, Poland) and Sunlu (Zhongshan, China) respectively. The properties of the materials are listed in Table 1.

The values of tensile strength and modulus provided by filament manufacturers were obtained for samples printed with 0.4 mm nozzle and 4 layers of outline/perimeter shells (Sunlu ePAHT-CF15). For tensile strength testing, the conducted research utilizes different, rectilinear/perimeter combination infill pattern, which is more suitable for high-volume 3D prints that are subjected to multidimensional loads.

The tests followed the BS EN ISO 527-2:2012 standard to ensure accuracy and consistency in evaluating the mechanical properties of the materials [37]. The ultimate tensile strength of these polymers was tested under a range of elevated temperatures to determine their performance in real-world conditions, particularly in applications where temperature resilience is crucial.

### 2.2. Test Specimens

The test specimens used for all strength tests were fabricated in accordance with the 1BA test specimen specification outlined in the BS EN ISO 527-2:2012 standard. The 1BA specimen is a standardized dog-bone shape, ensuring consistency across testing and allowing for reliable comparisons between materials. The specimens were 3D printed using the respective temperature-resistant polymer filaments, ensuring uniformity in size, shape, and orientation relative to the printing direction to minimize variability in test results.

### 2.3. Testing Procedure

The tensile tests were performed using a modified strength testing machine manufactured by VEB Thüringer Industriewerk Rauenstein (Schalkau, Germany), model ZE 200 157.11 capable of applying a uniaxial tensile force. The machine had a maximum load capacity of 200 kg, sufficient to measure the tensile strength of all tested materials. The pulling force from the load cell and the linear displacement were recorded by a dedicated data acquisition system. The machine has been modernized and upgraded to enable data acquisition via computer. The specimen was securely mounted in the machine’s grips, ensuring no slippage or movement during testing.

Temperature control was a key factor in these tests. To simulate real-world conditions, the test samples were heated from both sides using a controlled heating system. A generic 12V 15W silicone coated heater was used. A temperature controller Lumel RE16 (Zielona Góra, Poland), integrated with a PT100 resistor, was used to control the process ranging from 60 °C to 130 °C.

The present tensile tests were conducted under quasi-static loading at a single nominal strain rate in accordance with BS EN ISO 527-2:2012, using an electromechanical test machine. The resulting data allow a reliable determination of temperature-dependent Young’s modulus, yield stress, and ultimate tensile strength, which were used as input for static structural analyses. However, they are not sufficient to unambiguously calibrate advanced nonlinear viscoelastic or Eyring-type constitutive models, nor to perform time–temperature superposition, which would require multi-rate or dynamic mechanical tests over several decades of time or frequency. For the quasi-static, small-strain operating conditions of the impeller considered here, a temperature-dependent linear elastic description combined with experimentally measured strength limits was deemed adequate. The development and calibration of a fully time- and rate-dependent constitutive model for annealed ePAHT-CF15 is therefore left for future work.

#### 2.3.1. Specimen Preparation

**Figure 1:**
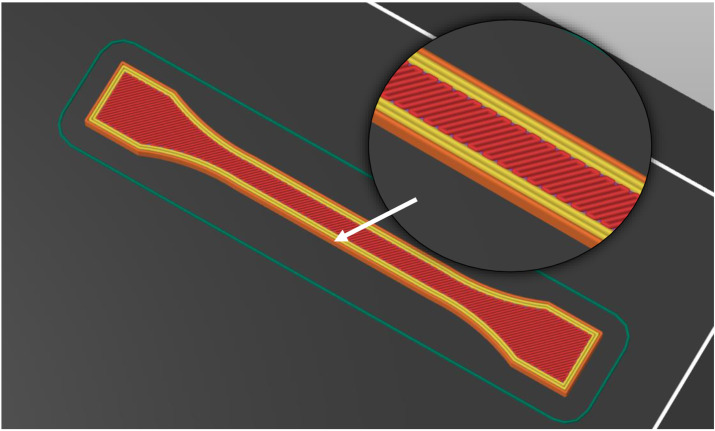
The 3D-printed specimen top layer view (orange—1 layer external perimeter, yellow—2 layers of internal perimeter, red—rectilinear infill).

The specimens were 3D printed to the dimensions specified by the BS EN ISO 527-2:2012 standard for the 1BA type. Each specimen was printed with the appropriate filament (ASA-X CF10, PA6-GF30, or ePAHT-CF15). The 3D printing of the specimen was carried out with three outer perimeters and a rectilinear infill set at 45° (Figure 1). This configuration was selected to better replicate the anisotropic material properties inherent in 3D-printed parts. Testing specimens composed exclusively of perimeters aligned in a single direction would yield unreliable results, as the strength along the printing path is considerably greater than the strength between layers.

#### 2.3.2. Annealing of ePAHT-CF15 Samples

In addition to the standard temperature testing, a batch of ePAHT-CF15 specimens was subjected to annealing. The POL-EKO (Wodzisław Śląski, Poland) SLN 53 STD heating chamber with a maximum temperature of up to 300 °C was used. As stated in [18], annealing improves the mechanical properties and temperature resistance of 3D prints. During annealing, internal stresses are reduced and reorganization of the polymer’s crystal chain structure occurs. This takes place when the material is heated to its glass transition temperature. Crystalline structures exhibit higher mechanical properties due to the better arrangement of atoms in the structure. Annealing involves heating the material to a certain temperature, holding it at that temperature for a certain amount of time, and then slowly cooling it down. The cooling takes place in a furnace. The sand in which the specimens were placed allowed for slow heating, shape preservation, and controlled cooling. Embedding in sand can reduce convective heat transfer and may introduce temperature gradients. To mitigate this, specimens were fully buried with uniform cover and held at the target temperature for a fixed soak time. Temperature uniformity inside the specimen was not instrumented. This is a limitation and should be verified in future work with specimen temperature mapping measurement with use of multiple thermocouple. The samples were heated in an oven at temperatures of 150 °C, 175 °C, and 200 °C for one hour. During the annealing process, no discoloration, microcracking, or embrittlement was observed. Annealing is expected to increase the crystallinity of the polymer, which may enhance its thermal resistance [32,38,39]. After annealing, the samples were tested in the same manner as the non-annealed specimens. Crystallinity was not quantified in this study (DSC/DMA/XRD measurements were not performed); the proposed mechanism is inferred from prior literature.

#### 2.3.3. Specimen Heating

**Figure 2:**
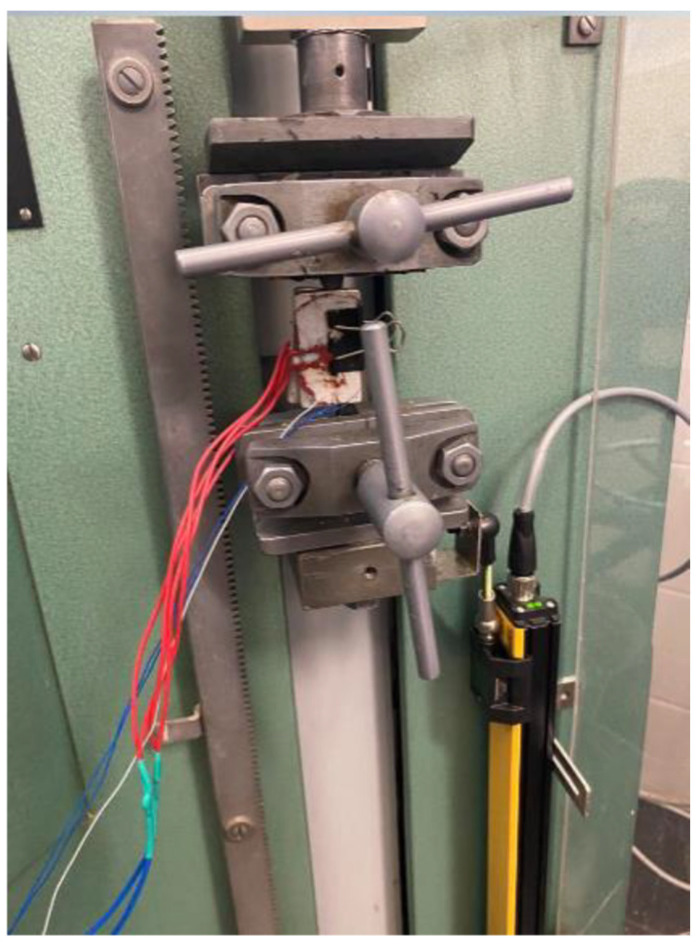
Experimental setup of the tensile test with heated specimen.

Prior to material properties examination, the specimen was positioned in the strength testing machine, and the temperature was set using the temperature controller. For the heater, a silicone heating pad glued to aluminium substrate was used. The power of the heater was equal to 6W. The PT100 resistor is located inside an aluminum heating plate attached to the sample. The specimen was then gradually heated from both sides to the desired testing temperature, which was held constant for 1 min after the set temperature had been reached. This ensures thermal equilibrium before starting the tensile test. The numerical solution of 1D heat equation with boundary condition of constant temperature heaters predicts that the equilibrium state for ePAHT-CF15 at 130 °C was reached after 19.6 s (temperature difference is smaller than 0.01% in the entire volume). An experimental setup was presented in Figure 2.

#### 2.3.4. Tensile Testing

Once the specimen reached the target temperature, the tensile testing machine applied a controlled tensile load. Specimens corresponded to BS EN ISO 527-2:2012 Type 1BA geometry, with gauge length *L*_0_ = 25 mm and initial grip separation *L* = 58 mm. The crosshead speed was *v* = 5 mm/min, corresponding to a nominal engineering strain rate of approximately ${\dot{\varepsilon }}_{nom}\;=\;$0.086 min^−1^ (8.6%·min^−1^, 1.4 × 10^−3^ s^−1^) when referenced to *L*. The selected crosshead speed provides quasi-static loading. In the impeller application, deformation is driven primarily by steady centrifugal load and slowly varying thermal expansion during operation. Therefore, quasi-static strain rates are representative for clearance-sizing analyses. Transient events like startup or shutdown may impose higher effective rates and are outside the present scope. For Young’s modulus determination, BS EN ISO 5272:2012 recommends a strain rate of 1%·min^−1^. This rate could not be used due to the short gauge length and limitations of the tensile testing machine.

During the test, force acting on load cell and linear transducer indication were recorded at a sampling rate of 10 samples per second (SPS). Engineering stress, engineering strain, ultimate tensile strength (UTS), and Young’s modulus were calculated using a Python 3.11 script.

#### 2.3.5. Repeatability and Validation

To ensure the reliability of the results, each filament was tested three times at each temperature point in the range 60–130 °C. The number of temperature levels depended on the filament. The annealed ePAHT-CF15 samples were also tested at the same elevated temperatures to compare their performance before and after annealing. Because PA-based composites are hygroscopic, to ensure the same parameters of the material, before the testing each specimen was dried in SUNLU FilaDryer S2 (Zhuhai, China) filament dryer for 24 h at 60 °C to relative humidity level of 12% and tested immediately after removal (sealed storage with desiccant between drying and testing). Moisture content and mass change after drying was not directly measured. Therefore, results correspond to a dried condition, and moisture sensitivity is treated as a limitation. Future research will focus on material properties of polymers with increased water content. Water absorption is another factor that can influence 3D−printed turbine impellers and requires separate study.

The average values of ultimate tensile strength and elongation at break were calculated and used for comparison across materials, temperatures, and annealing conditions. Repeatability was evaluated on an additional batch of 20 ePAHT-CF15 specimens annealed at 175 °C and tested at 100 °C under identical conditions (BS EN ISO 527-2:2012). The repeatability standard deviation for tensile strength was s_*r*,*UTS*_ = 1.89 MPa, corresponding to a coefficient of variation of *CV*_*UTS*_ = 4.82%. For Young’s modulus the repeatability standard deviation was *s*_*r*,__*YM*_ = 0.172 GPa (*CV*_*YM*_ = 6.17%). Using ISO 5725-2:2019 [40], the repeatability limits were *r*_0.90,__*UTS*_ = 2.33*s*_*r*,__*UTS*_ and *r_0.95,UTS_* = 2.77*s_r,UTS_* for tensile strength, and analogously for Young’s modulus. Thus, under repeatability conditions, the absolute difference between two results is expected to be below 4.4 MPa (90% confidence) or 5.2 MPa (95% confidence) for UTS, and below 0.40 GPa (90% confidence) or 0.48 GPa (95% confidence) for Young’s modulus. The obtained coefficients of variation are consistent with published data for FDM-printed fiber-reinforced polyamides [30,31].

## 3. Material Strength Testing Results

### 3.1. ASA-X CF10

**Figure 3:**
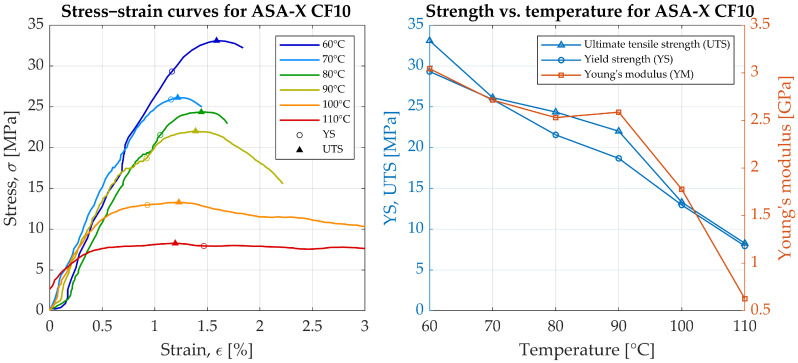
Experimental—results of ASA-X CF10 strength testing.

The tensile properties of ASA reinforced with carbon fiber were previously reported in [21]. This study investigates how the length distribution of carbon fibers affects the properties of ASA-based fiber-reinforced composites produced via large-format additive manufacturing (LFAM) using fused granular fabrication (FGF). Tests in aforementioned references were conducted at the room temperature. As a supplement to the results of previous studies, we present the results at elevated temperatures in Figure 3. The results were processed using a custom script to standardize the data. The highest ultimate tensile strength (UTS) of 33.1 MPa was recorded at 60 °C, with a Young’s modulus (YM) of 3.03 GPa and a yield strength (YS) of 29.4 MPa. As the temperature increased, a marked decline in the mechanical properties was observed. At 90 °C, below the material’s glass transition temperature, YM decreased to 2.59 GPa, YS dropped to 18.7 MPa, and UTS reduced to 22.0 MPa. At 100 °C, a significant change in material behavior was evident, with a higher strain of 9.37%, compared to strains of approximately 1.45% to 2.22% at lower temperatures. This suggests that the material transitioned into a highly elastic state, as indicated by a YM of 1.77 GPa, YS of 13.0 MPa, and UTS of 13.3 MPa. Notably, these values represent a substantial reduction from the cold-state datasheet values, with YS decreasing by 50 MPa and YM dropping by more than 50%.

As the temperature continued to rise, both UTS and YS decreased in the range from 60 °C to 100 °C, with UTS falling below 15 MPa at 100 °C. The UTS and YS values converged, indicating that the material’s highest stress occurred just after reaching the yield point, suggesting a rapid transition from plastic deformation to failure. Young’s modulus decreased with increasing temperature, except at 90 °C, where a slight increase in YM was observed. This behavior was observed on all three specimens that were tested at a given temperature.

At temperatures of 110 °C–120 °C, the material exhibited significantly higher strains of 18.46% and 40.01%, respectively. However, its strength properties were severely diminished, with UTS values of 8.3 MPa and 3.3 MPa. These reduced mechanical properties under thermal load render the material unsuitable for applications in district heating networks during the winter season, where maintaining strength at elevated temperatures is crucial.

### 3.2. PA6-GF30

**Figure 4:**
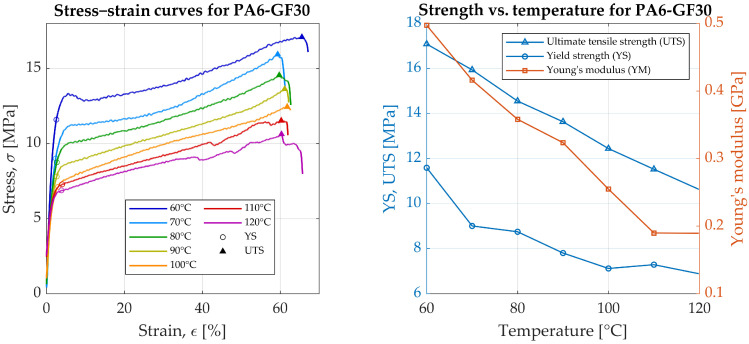
Experimental results of PA6-GF30 strength testing.

For PA6-GF30, the highest UTS of 17.1 MPa was observed at 60 °C, with a corresponding YS of 11.6 MPa, Figure 4. As the temperature increased, both UTS and YS values showed a decreasing trend. Young’s modulus ranged from 0.50 GPa at 60 °C to 0.19 GPa at 120 °C, indicating a substantial reduction in stiffness relative to ASA-X CF10, whose modulus was an order of magnitude higher. PA6-GF30 showed notable elasticity, with a maximum strain of 67.15% at 60 °C, and retained higher elongation at elevated temperatures, highlighting its higher resistance to thermal degradation compared to ASA-X CF10.

In comparison with the manufacturer’s datasheet at room temperature, PA6-GF30 exhibited significant reductions in mechanical properties when heated. Young’s modulus at elevated temperatures was at least 11 times lower than the cold-state value of 5.5 GPa. Yield strength was also markedly reduced, by at least six times. Since the specimen is printed with perimeter and rectilinear infill, the compared values might be different for the same printing setup. At 90 °C, the yield strength stabilized, with minimal change thereafter, and YM decreased steadily to 0.19 GPa by 120 °C.

### 3.3. ePAHT-CF15

**Figure 5:**
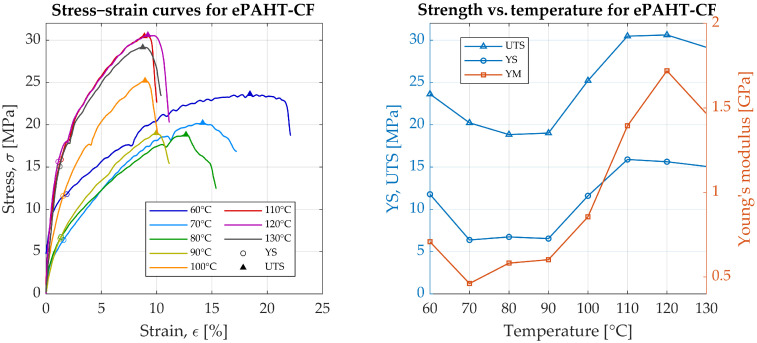
Experimental results of ePAHT-CF15 strength testing.

The averaged properties of perimeter/rectilinear infill specimen at room temperature are UTS = 89.1 MPa, YS = 74.6 MPa, YM = 6.6 GPa, and elongation at break equal to 2.2%. ePAHT-CF15 exhibited a gradual decrease in UTS with increasing temperature, from 23.6 MPa at 60 °C to 19.0 MPa at 90 °C (Figure 5). This reduction reflects a weakening of the material’s structural integrity as temperature increases. At 60 °C, YS was 11.8 MPa, but it exhibited substantial decrease as the temperature rose. At 70 °C, YS sharply dropped to 6.4 MPa, rebounded to 6.7 MPa at 80 °C, and decreased slightly to 6.5 MPa at 90 °C. Concurrently, the value of Young’s modulus showed the same nonlinear behavior, from 0.71 GPa at 60 °C, through 0.46 GPa at 70 °C, to maximal value of 1.72 GPa at 120 °C, and then dropped to 1.47 GPa at 130 °C.

Compared to the room temperature test, the mechanical properties of ePAHT-CF15 at 70 °C (worst case) were significantly reduced, with YM dropping 14 times and YS decreasing by 11.7 times. In the unannealed state, this material can be considered inconsistent and its properties are difficult to predict when used as a machine part operating over a wide temperature range. The increase in mechanical properties at temperatures higher than 90 °C can be connected with the changes in the internal structure of the material and the start of the annealing process.

### 3.4. Annealed ePAHT-CF15

**Figure 6:**
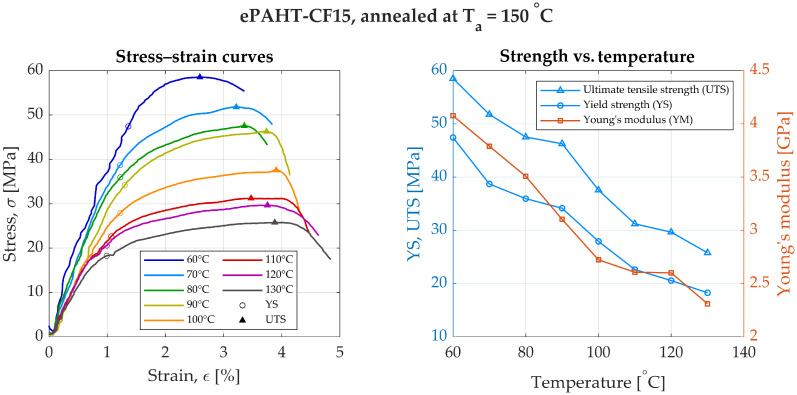
Experimental results of annealed ePAHT-CF15 strength testing (annealing temperature = 150 °C).

When subjected to annealing at 150 °C, ePAHT-CF15 exhibited improvements in mechanical properties. At 60 °C, YS increased to 47.4 MPa, while at 130 °C, it was equal to 18.3 MPa, Figure 6. A linear relationship of material strength drop with increasing temperature can be observed. UTS showed a general decrease with increasing temperature, from 58.5 MPa at 60 °C to 25.8 MPa at 130 °C. The maximum elongation at break was recorded as 4.84% at 130 °C. Young’s modulus decreased with temperature, starting at 4.1 GPa at 60 °C and dropping to 2.3 GPa at 130 °C. A reduction in strength properties compared to the cold-state values of unannealed specimen was 1.6 to 2.85 times lower for YM, and YS was reduced by a factor of 1.6 to 4.1 times in the entire temperature range.

**Figure 7:**
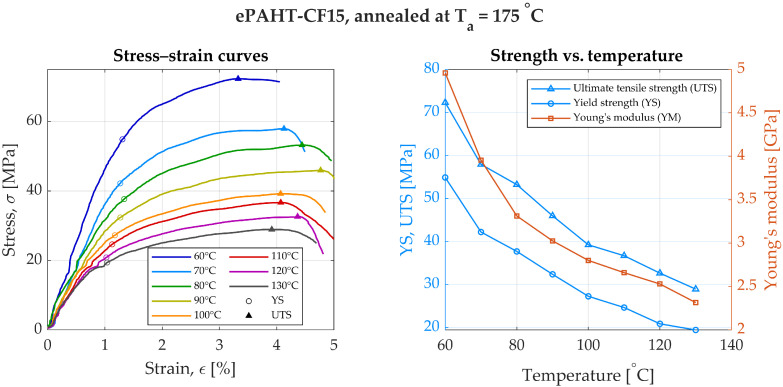
Experimental results of annealed ePAHT-CF15 strength testing (annealing temperature = 175 °C).

For specimens annealed at 175 °C, YS was 54.9 MPa at 60 °C and dropped to 19.4 MPa at 130 °C. UTS peaked at 72.3 MPa at 60 °C and decreased to 28.9 MPa at 130 °C, Figure 7. Young’s modulus at this annealing temperature was 4.96 GPa at 60 °C and decreased to 2.33 GPa at 130 °C. Compared to room temperature values of unannealed specimen, YM of ePAHT-CF15 annealed at 175 °C was 1.3 to 2.8 times lower, and YS was reduced by factors ranging from 1.6 to 3.8.

**Figure 8:**
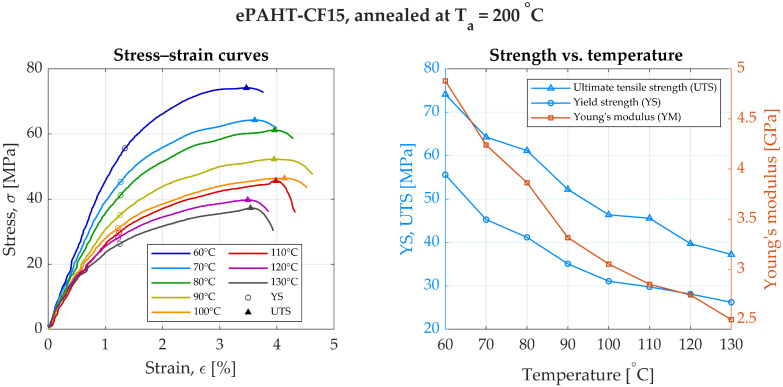
Experimental results of annealed ePAHT-CF15 strength testing (annealing temperature = 200 °C).

For specimens annealed at 200 °C, YS was 55.1 MPa at 60 °C and decreased to 26.5 MPa at 130 °C. UTS also exhibited a downward trend from 74.1 MPa at 60 °C to 37.3 MPa at 130 °C, Figure 8. Young’s modulus decreased from 4.89 GPa at 60 °C to a minimum of 2.47 GPa at 130 °C. The highest elongation (4.63%) was observed at 90 °C. Relative to the cold-state values of unannealed specimen, YM decreased by 1.1 to 2.2 times, while YS dropped by 3.1 to 6.5 times.

### 3.5. Strength Testing Summary

**Figure 9:**
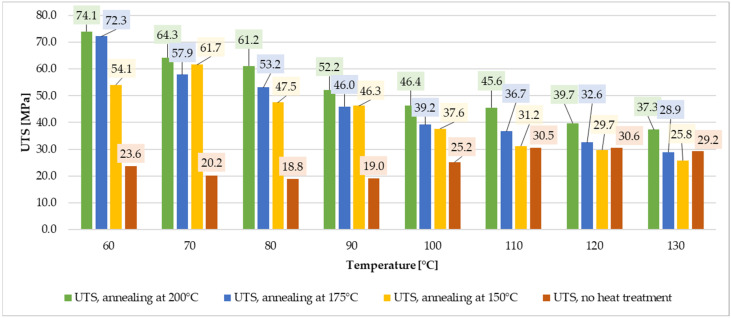
Comparison of UTS for ePAHT-CF15 values depending on annealing temperature or lack of heat treatment.

**Figure 10:**
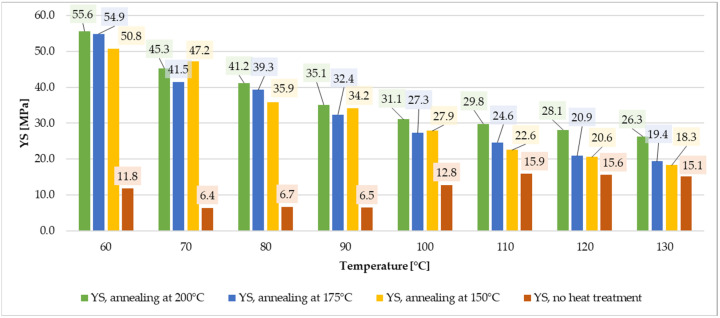
Comparison of YS for ePAHT-CF15 values depending on annealing temperature or lack of heat treatment.

**Figure 11:**
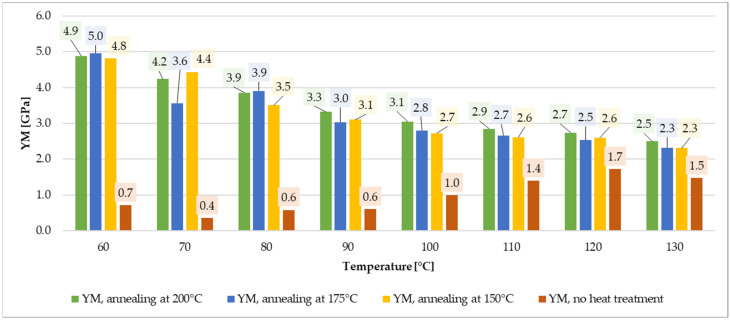
Comparison of YM for ePAHT-CF15 values depending on annealing temperature or lack of heat treatment.

The annealing process notably increased ultimate tensile and yield strength for lower temperature range (Figure 9 and Figure 10). The values for annealed specimens are more than three times higher than for non-annealed samples. Annealing at higher temperatures (175 °C–200 °C) enhanced thermal resilience for loads between 100 °C and 130 °C. Additionally, annealing at 200 °C resulted in a substantial increase in Young’s modulus, increasing material stiffness by factors ranging from 6.7 to 1.7, depending on the test temperature, Figure 11. These improvements make annealed ePAHT-CF15 the most suitable material for turbomachinery components out of all tested materials. Annealing at 200 °C provides the most significant mechanical properties rise out of all tested heat treatment temperatures.

In summary, ePAHT-CF15, particularly when annealed at 200 °C, demonstrates superior mechanical performance for high-temperature applications, making it the most appropriate choice for turbomachinery component design among the materials tested. During laboratory tests, higher annealing temperatures lead to slight rounding of sharp edges and loss of geometrical accuracy of the specimens, with a thermal/shape preserving mass (sand grains) attached to samples. Excessive softening due to the beginning of the 3D-print melting process prevents using annealing temperatures above 200 °C for high volume 3D prints such as turbine impellers. To ensure completeness of the measurement data and easier data processing for other researchers, tabulated values from Figure 9, Figure 10 and Figure 11 are included in the Appendix A, Table A1.

## 4. Pump as a Turbine Impeller Design

### 4.1. Impeller Design

**Figure 12:**
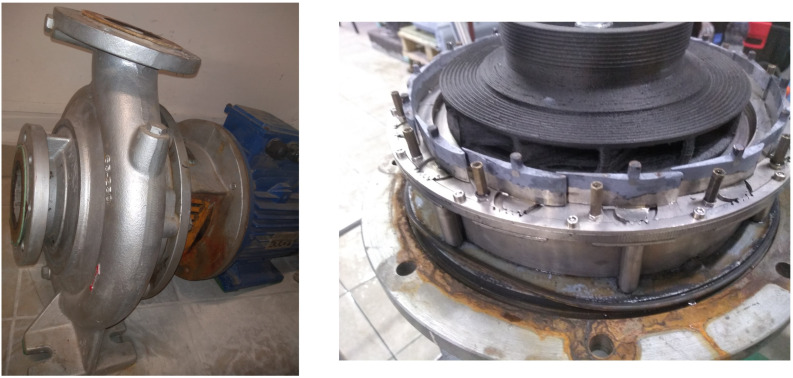
Hydro-Vacuum pump (model MVB65.250) and flow system with an inlet channel height of 16 mm.

**Figure 13:**
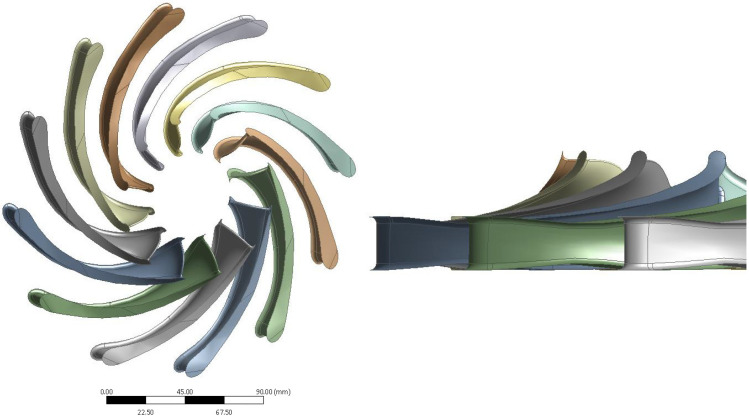
Geometry of blades prepared in BladeModeler and inlet choke reducing channel height.

Recent research conducted at the Faculty of Mechanical and Power Engineering [41] led to the development of a hydro-turbine based on a Hydro-Vacuum pump (Grudziądz, Poland, model MVB65.250) with a nominal flow rate of 68 $\frac{kg}{s}$ and a head of 30 m, as shown in Figure 12. Experimental and numerical analyses indicated that the original guide vane height of 9 mm resulted in a head loss of 2 m, which translates to approximately 10% of the overall efficiency loss of the machine. To improve this performance, the guide vane and rotor inlet height was increased to 16 mm, effectively increasing the inlet cross-sectional area and mitigating the head loss, as illustrated in Figure 13.

The design parameters for the impeller are as follows: the outer diameter of the radial inlet is *d*_1_ = 210 mm, the outlet diameter is *d*_2_ = 45 mm, and the volumetric flow rate is equal to *Q* = 65 $\frac{m^{3}}{h}$. The generator rotational speed is *n* = 1300 rpm, the guide outflow angle was *α* = 12.5° measured to a tangent line. This corresponds to approximately 80% of the guide vane opening. The guide vane outflow angle was used to derive inflow parameters for impeller.

The impeller geometry was produced by the ANSYS BladeModeler R23.0 module in combination with the Design Modeler. The generated geometry consisted of 12 blades, each with an inlet blade angle of 75.43°, Figure 13. To achieve smooth flow conditions, the blade angle curvature was designed to vary gradually along its length, avoiding abrupt changes that could adversely affect performance.

It is well-established that impellers operating as turbines tend to induce vortex structures at the inlet due to the relatively low inlet velocity. To address this issue and increase the inlet velocity, several design considerations were incorporated:

- The radius of the blade at the hub/shroud interface was set to 2 mm;
- The blade thickness was designed in a hydrofoil shape, starting at 4 mm at the leading edge (LE), expanding to a maximum thickness of 6 mm, and tapering to 1 mm at the trailing edge (TE);
- Blade rounding was applied at the inlet with a radius of 2 mm and at the outlet with a radius of 1 mm. This design feature is known to enhance turbine efficiency by reducing flow separation and minimizing turbulence [42];
- A channel choke with a height of 12 mm was implemented at the radial inlet;

These design modifications, which include increasing the number of blades, narrowing the inlet, and thickening the blade at the leading edge, were intended to accelerate the flow velocity and reduce the vortex effect at the inlet. These changes are expected to improve the overall efficiency of the turbine operation by optimizing the fluid dynamics at the impeller inlet.

For the CFD-based flow analysis, the impeller geometry shown in Figure 13 was used. It was optimized on a coarse grid with the use of a multi-parameter genetic algorithm engine. Optimization was performed on three spanwise locations, where the input parameters were blade angles, thickness, and channel choke size. To reduce computational time, a single periodic axisymmetric blade channel was evaluated. The analysis was conducted using ANSYS CFX R23.0 software. To simplify the problem, simulation through the sealing gap and the pressure gradient calculation on the outer front disc was excluded. Adding additional loads would require complex leakage calculations, which were beyond the scope of this study.

### 4.2. Mesh Generation

**Figure 14:**
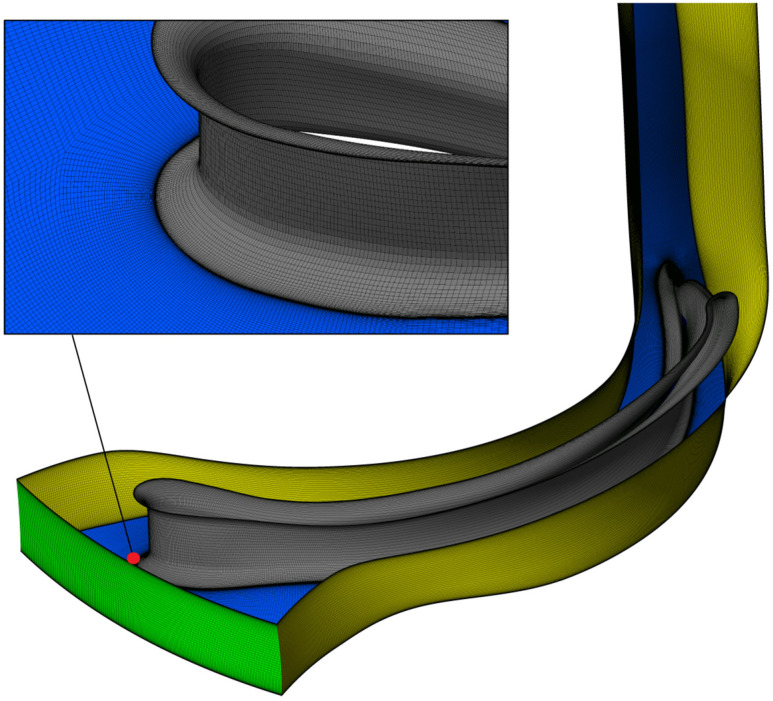
Mesh generation of periodic impeller channel for the flow analysis (green surface–impeller inlet, red surface–impeller outlet).

The mesh for the hydro-turbine was generated using the ANSYS TurboGrid R23.0 module, which enables the rapid creation of high-quality hex-meshes with inflation layers in areas where velocity gradients are significant. The coarse, 100,000 element grid used for optimization of impeller geometry was refined to approximately 18 million elements, Figure 14. This allowed for capturing the pressure field most accurately. Mesh quality metrics are as follows:

- Minimum orthogonality angle was equal to 31°.
- Maximum edge length ratio was lower than 200.
- Element volume ratio was lower than 2.6.
- Skewness was better than 0.81.
- First element height was equal to 4 μm.

High quality numerical grid was used to perform simulation with the use of high-resolution schemes both for advection and turbulence (CFX Solver Theory Guide states it is bounded, second-order upwind biased discretization). The mesh was modified relative to the initial setup to improve near-wall resolution. Additionally, the first-layer thickness was adjusted to target y^+^ ≈ 1. The domain design was modified with respect to the impeller design. The outlet of the domain was moved further from the impeller exit to prevent oscillations, reverse flows, and satisfy zero gradient boundary condition at the exit.

### 4.3. Boundary Conditions and CFD Simulation

A single blade was modeled with rotationally periodic boundary conditions. At the outlet, 0 Pa gauge pressure was applied. The inlet boundary conditions were based on the mass flow rate of 1.5 $\frac{kg}{s}$. Based on the inflow angle and flow rate, the velocity components were defined as follows: the axial component of the cylindrical velocity was set to 0 $\frac{m}{s}$, the radial component to −1.71049 $\frac{m}{s}$, and the tangential (θ) component to 7.71554 $\frac{m}{s}$. The rotational speed of the channel was equal to 1300 rpm. The flow was considered incompressible, with water as the working fluid. Due to the use of a fine mesh resulting in low values of y+~1, the SST turbulence model and double-precision arithmetic were employed for the simulation. Periodic (cyclic) boundary conditions enforce flow continuity between adjacent passages for an ideally periodic 12-blade impeller; blade-to-blade variations were therefore not considered.

**Figure 15:**
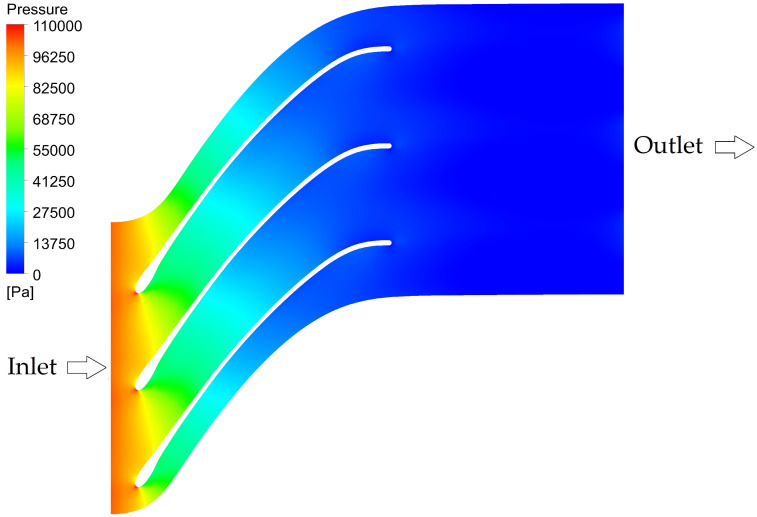
Pressure distribution at midspan of the impeller.

The pressure distribution on the impeller was used for calculating hydrodynamic load and performing structural analysis, Figure 15. The total wall clock computation time of simulation was 6.58 × 10^4^ s (650 iteration, 40 threads on dual Xeon E5-2696v4 CPU). The numerically estimated efficiency of the impeller was 90.8% (internal blade system only). Components such as surface roughness, sidewall gaps, and disk friction, which affect the overall turbine efficiency, were not included in the calculation of the impeller efficiency. The hydraulic power for this geometry is 2435 W, and the shaft power is 2212 W. The available net head is 11.31 m. The simplifications used for calculation might affect the real impeller performance. Nevertheless, the results indicate that the designed impeller is suitable for the operating point. It should be noted that the flow calculations are only used to estimate the flow field and are not the primary goal of this research work.

## 5. Structural Analysis

### 5.1. Problem Formulation

**Figure 16:**
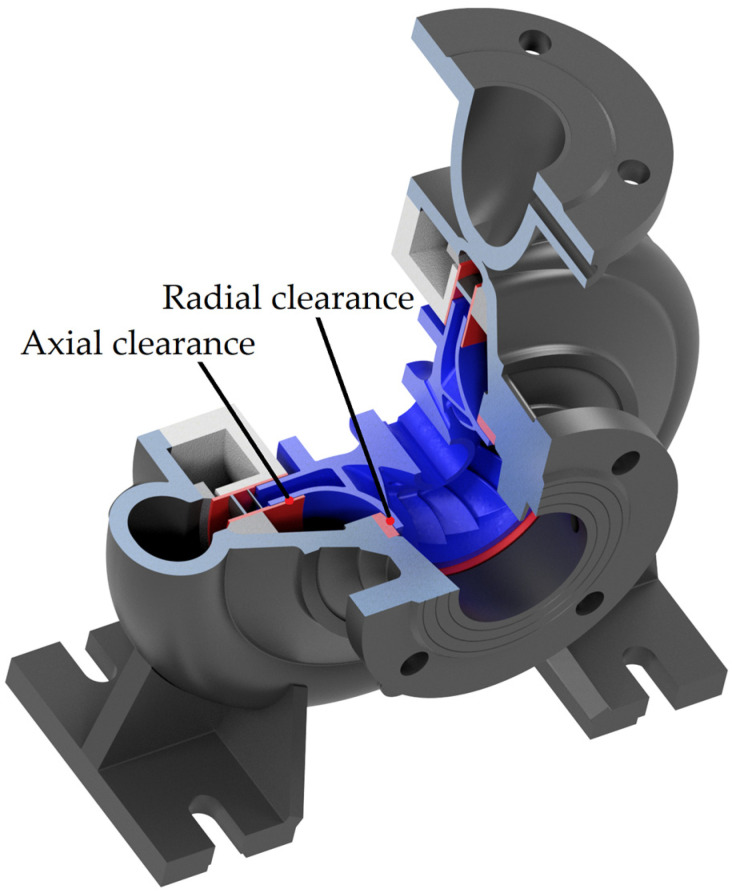
Shroud and hub impeller-volute clearance (blue—impeller, red—seal surfaces, grey—volute casting).

To ensure optimal impeller performance, the clearances between the hub and cover, the tip clearance, and the clearance between the shroud and bottom ring must be precisely defined, as illustrated in Figure 16. Minimizing the axial clearance is particularly important, as it reduces volumetric losses, thereby enhancing overall efficiency [43].

The prior PAT impeller model [41] exhibited excessive deformation, leading to unwanted frictional contact with the seal surface. As a part of the design, it is essential to conduct a numerical structural assessment to quantify the deformations of the impeller and determine the required clearances to prevent such issues.

### 5.2. Geometry Preparation and Boundary Conditions

**Figure 17:**
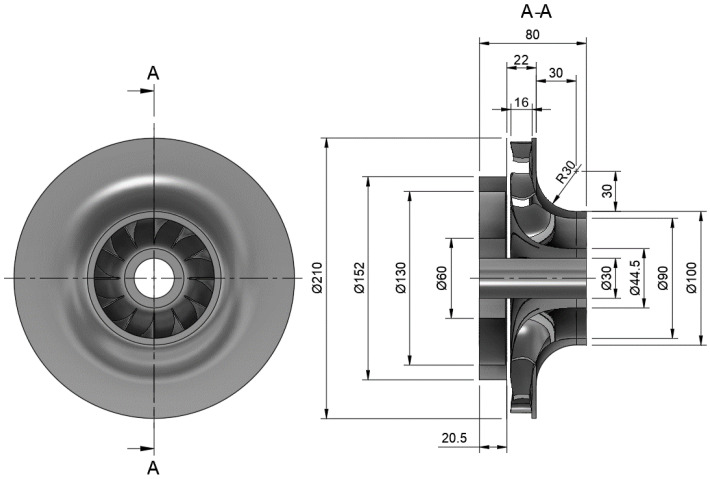
PAT impeller model prepared for structural analysis.

The 3D geometry for structural analysis was developed using Design Modeler R23.0 (Figure 17). To reduce global mesh size, the geometry was intentionally simplified by removing the fillet between the hub, the shroud, and the blading system. Two materials operating at temperatures of 70 °C and 120 °C were considered for analysis: PA6GF30 and ePAHT-CF15 (annealed at 200 °C). Material properties were obtained from tensile strength tests. Structural simulations were performed using the ANSYS Static Structural module. The Static Structural module was used in linear-elastic mode to estimate small-deflection clearance changes under service loads. This is appropriate for first-order gap sizing when strains remain below the onset of yielding. The tensile curves obtained during the experiment show temperature-dependent nonlinearity and yield. Therefore, results should be interpreted as conservative elastic estimates. A fully nonlinear (elasto-plastic/viscoelastic) model would require calibrated temperature and rate-dependent constitutive data like multiple strain rates, creep, or relaxation, which was beyond the scope of this work. For each simulated temperature of operation, separate strain–stress curve dependance for the linear-elastic model was employed.

**Figure 18:**
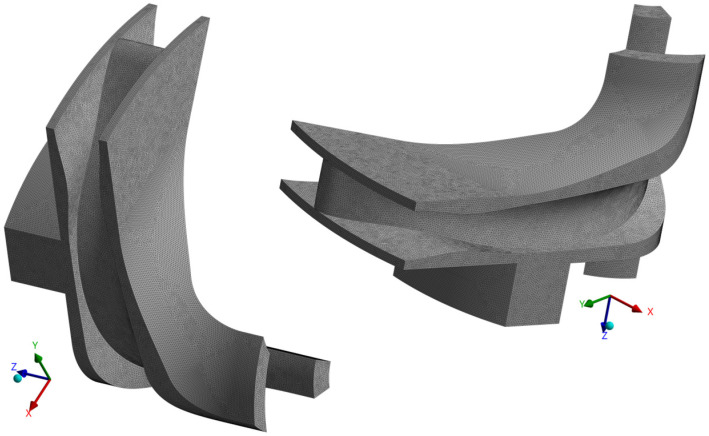
Mesh used for structural analysis of PAT impeller.

The numerical surface mesh consisted of triangular elements with a constant size of 0.6 mm. It has 1,979,736 elements and 2,789,467 nodes, Figure 18. The domain is assumed to be axisymmetric with respect to the *Z*-axis (a 1/12th slice). Cyclic symmetry boundary conditions were applied between the two radial cut faces using an ANSYS Mechanical Cyclic Region; the sector angle was 30° and the axis of rotation was the shaft axis (Z). Cyclic symmetry enforces periodic displacement continuity between sector faces, capturing inter-blade coupling under pitch-periodic loading. Blade-to-blade variations arising from manufacturing scatter, damage, or non-uniform loading are not captured in a single-sector model and would require a full 360° model or multi-sector perturbation analysis.

In order to minimize discretization errors associated with the finite element method, calculations were performed for different mesh sizes. The mesh was gradually refined for element sizes ranging from 5 mm to 0.6 mm. An element size of 0.6 mm can provide a mesh-independent solution with reasonable calculation time. The total deformation change in mm was of the order of 10^−2^ mm with respect to coarser mesh (2 mm).

The impeller was fixed on the shaft at a diameter of ∅30 mm. The temperature load was modeled as the environmental temperature at which the component operates. The impeller was at constant, elevated temperature, without gradients that could cause thermal stress. For the simulation, thermal expansion in deformation analysis was neglected, but is discussed in Section 5.4. A rotational speed was set to 1300 rpm. The pressure distribution was mapped from the flow analysis as the pressure acting on the impeller walls and blade.

### 5.3. Structural Strength Simulation Results 

The impeller, subjected to pressure load, exhibits a maximum von Mises stress of 4.9 MPa. For all investigated cases, the stress values remain well below the material’s yield strength, ensuring safe operational conditions. Specifically, the stress is at least 1.4 times lower than the yield strength of PA6-GF30, the material with the lowest strength among those considered, at a thermal load of 120 °C. The stress is 1.8 times lower than the yield strength of PA6-GF30 at 70 °C, while for ePAHT-CF15, the maximum stress is 9.3 times lower at 70 °C and 5.8 times lower at 120 °C. The peak stress occurs at the blade–shroud interface, where stress concentration is localized. However, in an actual design, this stress concentration would be eliminated by means of the fillet at the blade–shroud and blade–hub connection.

**Figure 19:**
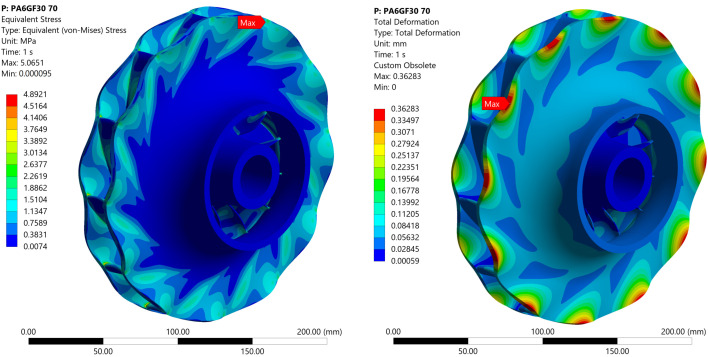
Equivalent von Mises stress and total deformation for an impeller made of PA6-GF30 operating at 70 °C.

For PA6-GF30 operating at 70 °C, the maximum total deformation is 0.36 mm, located at the impeller tip, Figure 19. The deformation was magnified for better visualization of the impeller operation under load. The corresponding equivalent elastic strain is 0.9%, which exceeds the material’s plastic deformation threshold. This suggests that the impeller could experience local permanent deformation under operational conditions.

**Figure 20:**
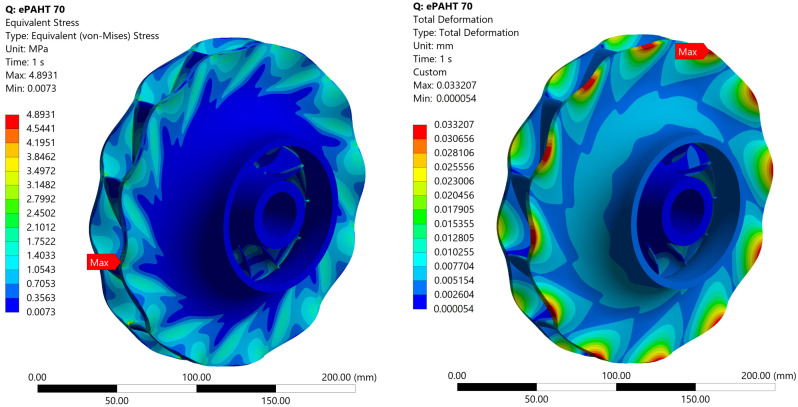
Equivalent von Mises stress and total deformation for an impeller made of ePAHT-CF15 operating at 70 °C.

For ePAHT-CF15 operating at 70 °C (Figure 20), the stress distribution remains identical to that of PA6-GF30, as the pressure load and rotational speed are unchanged. However, the maximum total deformation is significantly lower, measuring 0.033 mm, which is approximately an order of magnitude smaller than the deformation observed in PA6-GF30 under identical conditions. The equivalent elastic strain of ePAHT-CF15 is equal to 0.08%, which is an order of magnitude lower than that of PA6-GF30, indicating substantially higher material rigidity. Results for a case with post-printing heat treatment material clearly indicate that enhanced rigidity is crucial for ensuring the structural integrity and operational performance of the impeller, particularly under sustained loading.

**Figure 21:**
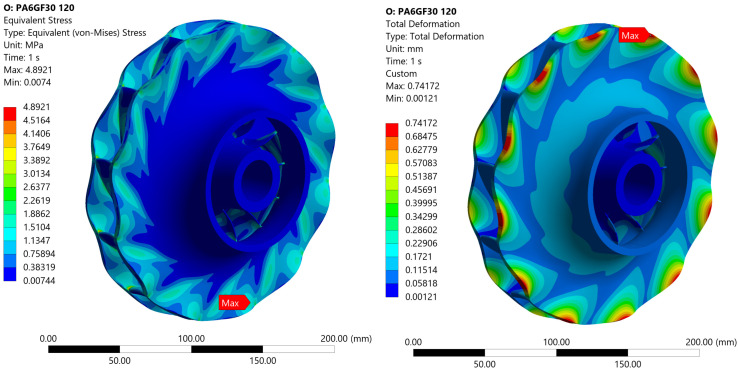
Equivalent von Mises stress and total deformation for an impeller made of PA6-GF30 operating at 120 °C.

At 120 °C, the maximum von Mises stress in the PA6-GF30 impeller is 1.4 times lower than the yield strength of the material, Figure 21. The total deformation increases to 0.74 mm, more than double that observed at 70 °C. The equivalent elastic strain increases to 1.8%, reflecting a 0.9 percentage point increase compared to the lower operating temperature, suggesting a more significant elastic deformation in the material at elevated temperatures.

**Figure 22:**
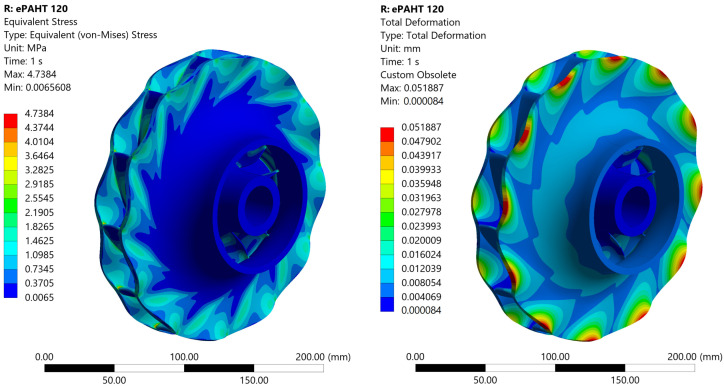
Equivalent von Mises stress and total deformation for an impeller made of ePAHT-CF15 operating at 120 °C.

For the ePAHT-CF15 impeller at 120 °C, the maximum von Mises stress is 2.64 MPa, which is 5.7 times lower than the yield strength of this material at 120 °C, Figure 22. The total deformation is 0.052 mm, which is more than 14 times smaller than the deformation observed in the PA6-GF30 impeller at the same temperature. This highlights the significantly greater rigidity and thermal stability of ePAHT-CF15. The equivalent elastic strain is limited to 0.1%, demonstrating that the ePAHT-CF15 impeller exhibits negligible plastic deformation under the given loading conditions. This behavior underscores the material’s superior strength and suitability for high-performance applications at elevated temperatures.

In summary, the structural analysis confirms that the ePAHT-CF15 material offers superior rigidity, thermal resistance, and overall strength when compared to PA6-GF30, making it a more suitable choice for the impeller design, particularly under high-temperature operating conditions.

### 5.4. Axial Sealing Gap Adjustment

**Figure 23:**
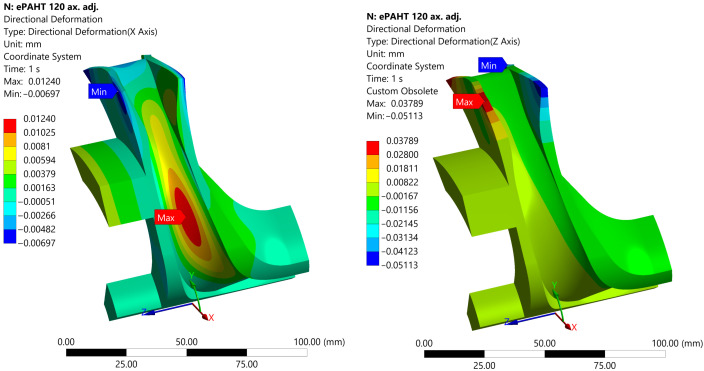
Deformation along *Y*-axis and *Z*-axis of ePAHT-CF15 impeller operating at 120 °C.

One of the most important aspects of correct machine operation is definition of the required clearances. The analysis focused on an impeller made from ePAHT-CF15 operating at 120 °C. The resulting deformations in the cylindrical system of coordinates along the *Y*-axis (radial) and *Z*-axis (axial) are presented in Figure 23.

In the radial direction, the maximum deformation of the shroud was 0.27 μm, while the hub experienced a radial deformation of 0.47 μm. This is mainly caused by centrifugal force. The central part of the impeller blade suffered the most deformation, 12.4 μm. In the axial direction, the hub deformed by a maximum of 37.9 μm, while the front shroud exhibited a maximum deformation of 51.1 μm in the opposite direction. These deformations, particularly in the axial direction, could influence the axial gap and overall clearance between components, potentially affecting the impeller’s sealing performance.

Given the dimensional tolerance of ±0.25 mm typical for high-quality FDM printing, achieving the precise clearances required may not be feasible. To enhance run-out tolerance, it is recommended to use an adapter made of steel that will couple the shaft and the impeller. After annealing, when some deformation can occur, the entire assembly of metal insert and the impeller should be turned true on the lathe with a carbide-lapped insert to achieve a smooth surface, accurate dimensions, and rotary movement precision.

Another aspect that influences the gap sizing tolerance is thermal expansion of polymers. It should be noted that the cast iron, stainless steel, and polymers can differ in the coefficient of thermal expansion (CTE) by an order of magnitude. For cast iron and stainless steel, the CTE is typically from 9 to 12·10^−6^/°C. Polymers, on the other hand, can exhibit extremely different values. Measured according to ISO 11359-2:2021 Standard [44], the molded PA6 CTE is typically equal to 80−110·10^−6^/°C, glass fiber-reinforced PA6 is 20·10^−6^/°C, and special grades like Luvocom PAHT-CF 9742 BK can be as low as 5·10^−6^/°C. After determining the operating temperature, thermal expansions, water absorption testing, and including the influence of additional material impregnation methods, it is possible to individually determine the technological clearances.

Apart from sodium silicate mentioned in the introduction, high-temperature electro-insulating varnish could remedy the absorption problem; however, more lab testing should be done. As for sodium silicate, it is easy to apply, but 24-h pressurized CO_2_ hardening of the entire impeller 3D print volume was not successful during our experiments. High-temperature electro-insulating varnish is hardened by temperature. During application at room temperature, the viscosity is very high. When heated it becomes thin, so removing excess coating/residues might be a challenge.

Consequently, mainly because of cavitation risk and water absorption, it is recommended to set the radial clearances at the tip and hub not smaller than 0.30 mm. For the axial clearances, values of 0.45 mm for the rear shroud and 0.6 mm for the front shroud are suggested as a starting point (dimensioning in dry state). These proposed clearances account for manufacturing tolerances while ensuring that the impeller operates within the required parameters for effective sealing and minimizing performance losses. The suggested values strike a balance between theoretical design requirements and practical manufacturing constraints. CTE of the printed composites was not measured in this study. Therefore, clearance recommendations should be treated as preliminary until ISO 11359-2:2021 measurements are performed for the printed material.

## 6. Conclusions

This investigation introduces a novel approach to additive manufacturing of hydro-turbine impellers, emphasizing the development and application of advanced 3D-printing materials tailored for high-temperature operational environments within the energy sector, particularly for district heating systems. This study systematically evaluates the mechanical performance of three candidate materials—ASA-X CF10, PA6-GF30, and ePAHT-CF15—under elevated temperature conditions (60 °C to 130 °C), with a particular focus on their suitability for functional, load-bearing impeller components. A key innovation lies in the application of a heat treatment process—specifically annealing at 200 °C—to enhance the mechanical properties of ePAHT-CF15. This thermal post-processing resulted in a remarkable increase in stiffness, with the Young’s modulus at 70 °C reaching 4.25 GPa, representing a 3.87 GPa improvement over untreated samples. Similarly, at 90 °C, the Young’s modulus was elevated to 3.32 GPa, surpassing the non-annealed material by 2.83 GPa. When benchmarked against traditional materials such as ASA-X CF10 and PA6-GF30, annealed ePAHT-CF15 demonstrated superior strength characteristics, notably exhibiting a yield strength more than four times greater than PA6-GF30.

Simulation results reveal that impellers constructed from annealed ePAHT-CF15 exhibit drastically reduced deformation—by a factor of ten at 70 °C and fourteen at 120 °C—highlighting its potential for reliable operation under thermal and mechanical loads. These findings are critical for maintaining precise operational clearances, thereby ensuring efficiency and longevity of the turbine components.

In conclusion, this research provides valuable insights into the application of 3D printing in the energy sector, particularly for energy recovery in district heating systems. The key findings of the study are as follows:

- Among ASA-X CF10, PA6-GF30, and ePAHT-CF15, ePAHT-CF15 is the most suitable material for 3D-printed PAT impellers operating in district heating systems;
- Material selection is primarily influenced by its stiffness and heat deflection behavior, with a focus on achieving high values of Young’s modulus and heat deflection temperature;
- Annealing ePAHT-CF15 significantly improves its strength properties;
- For tests at 60 °C, the Young’s modulus of annealed ePAHT-CF15 increased seven-fold, its yield strength five-fold, and its ultimate tensile strength is tripled. At 120 °C, it also showed significant improvement of mechanical strength;
- Based on the technical specifications provided, the impeller geometry for the MVB65.250 hydro-turbine pump was prepared, and a flow analysis was conducted to obtain the pressure distribution across the shroud and blade;
- Structural analysis confirmed that ePAHT-CF15 exhibited the lowest deformation, with values of 0.033 mm at 70 °C and 0.052 mm at 120 °C;
- The deformation results allowed for precise definition of operational and technological clearances;
- Future work will focus on long-term hydro-thermal aging and combined fluid–structure simulations to validate the performance of the printed impellers in real PAT operation.

## Figures and Tables

**Table 1:** Properties for selected materials.

Properties	ASA-X CF10	PA6-GF30	ePAHT-CF15
Tensile strength, MPa	79	80	173
Tensile modulus, MPa	7580	5500	−
Flexural modulus, MPa	−	4500	5612
Density, g/cm^3^	1.1	1.3	1.4
HDT 0.45 MPa, °C	90 °C	180 °C	190 °C
Printing temperature	235–260 °C	250–280 °C	240–300 °C

## Data Availability

The original contributions presented in this study are included in the article. Further inquiries can be directed to the corresponding author.

## Abbreviations

Most common abbreviations used in this manuscript are as follows:

3D	Three-dimensional
AM	Additive manufacturing
CTE	Coefficient of thermal expansion
FDM	Fused deposition modeling
FFF	Fused filament fabrication
HDT	Heat deflection temperature
UTS	Ultimate tensile strength
YS	Yield strength
YM	Young’s modulus
CFD	Computational fluid dynamics
PA	Polyamide
PA6-GF30	Polyamide-6 reinforced with 30% glass fiber
ASA	Acrylonitrile styrene acrylate
ePAHT-CF15	High-temperature PA-based filament with 15% carbon fiber

## Appendix A

**Table A1:** Comparison of the strength data for ePAHT-CF15 after and without annealing.

Temperature [°C]		60	70	80	90	100	110	120	130
**Annealing at 200 °C**	**UTS [MPa]**	74.2	64.3	61.1	52.2	46.3	44.7	39.7	37.2
	**YS [MPa]**	55	45.2	41.4	35.1	31.1	30	28.3	26.5
	**YM [GPa]**	4.9	4.2	3.9	3.3	3.05	2.9	2.74	2.5
**Annealing at 175 °C**	**UTS [MPa]**	72.3	57.8	53.2	45.7	39.2	36.7	32.5	28.9
	**YS [MPa]**	54.7	41.6	39.4	32.4	27.3	24.5	21.3	18.2
	**YM [GPa]**	5	3.6	3.9	3.0	2.8	2.7	2.5	2.3
**Annealing at 150 °C**	**UTS [MPa]**	54.2	61.8	47.4	46.2	37.2	31.2	29.6	25.8
	**YS [MPa]**	50.4	47	36	34.9	27.8	22.7	20.9	18.4
	**YM [GPa]**	4.8	4.4	3.5	3.1	2.7	2.6	2.6	2.3
**No heat treatment**	**UTS [MPa]**	23.6	20.2	18.8	19	25.2	30.5	30.6	29.2
	**YS [MPa]**	11.8	6.4	6.7	6.5	12.8	15.9	15.6	15.1
	**YM [GPa]**	0.71	0.35	0.58	0.6	1	1.4	1.7	1.47
